# Osseointegration of Titanium Implants in Onlay of Cerament™, a New Ceramic Bone Substitute

**DOI:** 10.3390/jfb7010002

**Published:** 2016-01-07

**Authors:** Anna Truedsson, Jian-Sheng Wang, Pia Lindberg, Gunnar Warfvinge

**Affiliations:** 1 Department of Oral Pathology, Faculty of Odontology, Malmö University, Malmö SE-205 06, Sweden; pia.lindberg@mah.se (P.L.); gunnar.warfvinge@mah.se (G.W.); 2 Department of Orthopedics, Lund University Hospital, Lund SE-221 85, Sweden; jian-sheng.wang@med.lu.se

**Keywords:** animal model, bone substitutes, histological analysis, osseointegration

## Abstract

The purpose was to investigate whether a new biphasic and injectable ceramic bone substitute Cerament™ that rapidly remodels to bone, may contribute to the retention of titanium implant screws during the healing period, and to analyze the pattern of bone formation around titanium implants.Titanium screws were implanted in rat tibiae and embedded with or without Cerament™ on the cortical surface. Torsional resistance was measured after 1 day, and after 6 and 12 weeks. Implant areas without bone substitute were analyzed histologically for comparison. The torsional resistance increased over time as the screws were osseointegrated. There was no difference in resistance between screws embedded in the bone substitute and control screws. The bone apposition was more pronounced on the proximal side of the screw than on the distal side. Cerament™ is capable of conducting bone growth from a cortical bone surface. The newly formed bone in this application does not significantly add to the osseointegrative strength of the implant screw, as measured by torque resistance, during the first 12 weeks.

## 1. Introduction

Dental implants require sufficient retention and often call for augmentation of the alveolar ridge, either as buccal onlays or as augmentation of the floor of the maxillary sinus. Up to now, autologous bone from the jaw or the iliac crest has been the golden standard grafting material when new bone is needed [1,2]. However, a significant drawback of bone grafting is the risk of donor site morbidity [3,4] and in a quest for alternatives, several ceramic bone substitutes have been introduced [5,6,7]. Biomaterials have thus to some extent reduced the need for bone autografts but as yet, only few have been sufficiently evaluated in order to constitute a reliable option [8].

The outcome measure of bone augmentation is often success of the dental implant in a longer perspective. On the other hand, in experimental settings, retention of implants has been tested with various mechanical tests including pull-out and torque tests. Pull-out tests are more widely used and it has been shown that bone substitutes may add strength to osteoporotic bone [9,10]. Torque tests have been used to investigate if onlay bone grafts may add strength to the integration between bone and dental implants and it has been shown that dental implants anchored to a rabbit cortical bone surface with onlay has a greater removal torque resistance (RTQ) after 24 weeks than control implants without onlay [11,12]. An animal study with various types of grafting material and implants displayed no alteration in torque removal value but differences in bone to implant contact percent [13]. Removal torque is measured in a three-dimensional situation whereas the histological bone to implant contact may be calculated in two dimensions. Many animal studies have been done with different implant surface in the purpose to measure correlation between removal torque and bone to implant contact/bone volume [14,15,16].

Cerament™ is a new ceramic bone substitute that was originally designed to treat spinal fractures and to fill bone voids. The substitute was used in a recent study of 33 patients who underwent percutaneous vertebroplasty after osteoporotic and/or traumatic vertebral fractures. Radiological and clinical outcome was assessed by radiography, CT, and MRI, and 12 months after surgery, the fractures were stable and new bone formation visible [17]. It is a biphasic mixture of calcium sulphate hemi-hydrate (CSH) and hydroxyapatite (HA) that has good compressive strength [18]. CSH is a bone substitute with well-documented ability to support healing in bone defects [19] but the usefulness of pure CSH in a clinical setting is limited due to its low resistance to resorption [20]. In contrast, hydroxyapatite (HA) has both good mechanical strength and resistance to resorption as well as osteoconductive properties [6]. In Cerament™, the CSH component is readily dissolved leaving HA as a three-dimensional osteoconductive matrix that allows ingrowth of osteogenic cells and vessels. Hence, Cerament™ has many features that are desirable in oral and maxillofacial reconstructive surgery.

Experimental evaluation of bone substitutes have primarily been conducted through analysis of bone ingrowth into drilled defects and other artificial bone voids. Clinically however, the surgeon is often confronted with a need to augment rather than fill and there is a need for studies on bone substitute onlays. In an experimental onlay model, we have previously shown that Cerament™ may guide bone to augment a cortical bone surface in rats, detailing histological features of the bone remodeling [21]. The purpose of this study was to investigate if Cerament™, and the newly formed bone within it, may also contribute to the retention of implants during the healing period. We have also histologically studied the bone healing reaction around implants in rat tibia.

## 2. Results and Discussion

### 2.1. Biomechanical Analysis

**Figure 1:**
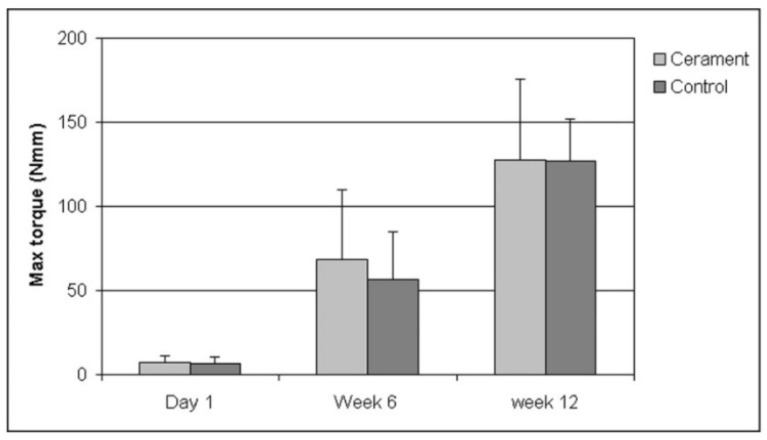
Torque resistance of titanium screws implanted in rat tibia after different periods of osseointegration. The bars denote mean maximum torque resistance from eight measurements (seven at day 1). Thin lines represent 1 SD.

The biomechanical test was performed for all time periods (1 day, 6 and 12 weeks). The maximum RTQ increased from 1 day to 6 weeks and further to 12 weeks (*p* < 0.001; Figure 1). However, there were no significant differences between the RTQ values of titanium screws implanted with Cerament™ and the controls without, at any time period.

**Figure 2:**
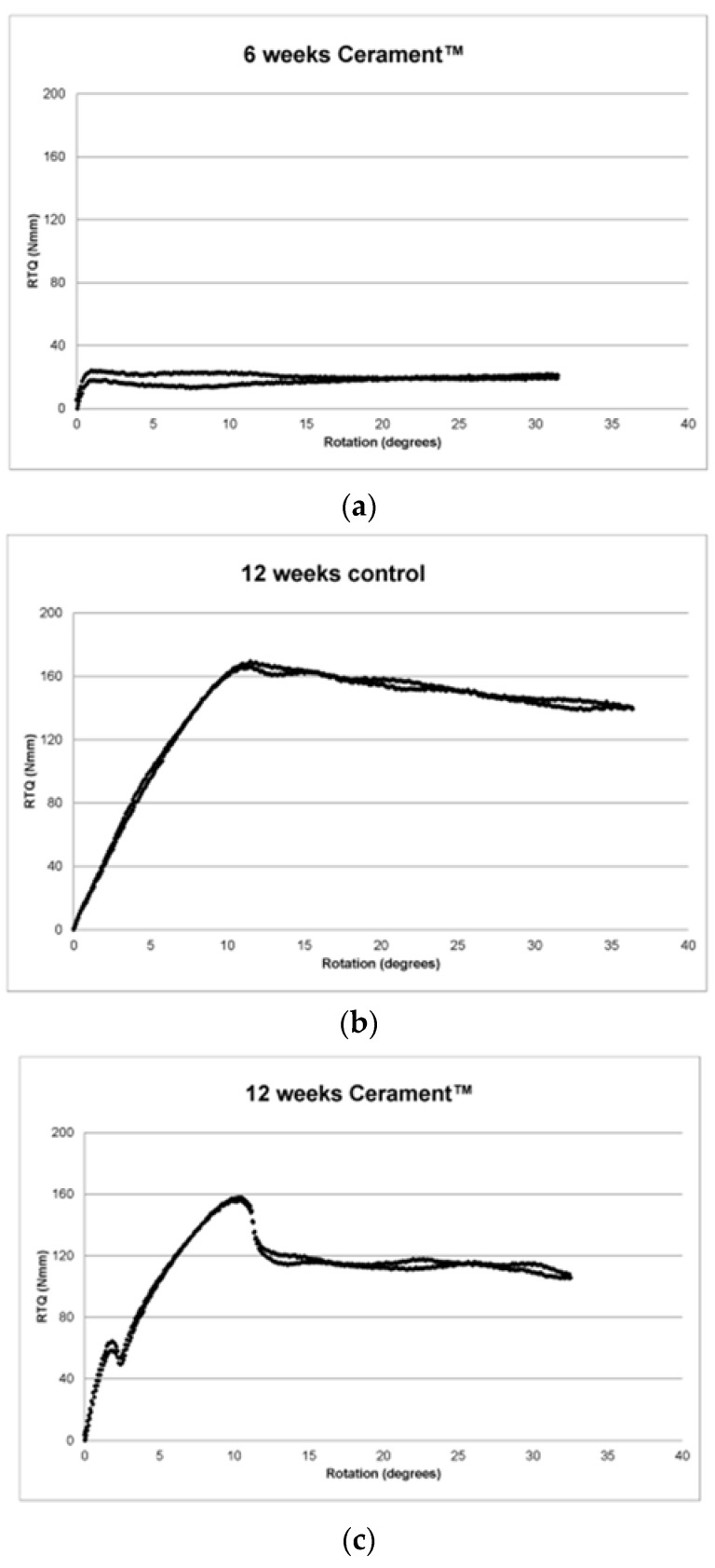
(**a**) RTQ 6 weeks Cerament™; (**b**) RTQ 12 weeks control; (**c**) RTQ 12 weeks Cerament™.

The RTQ plots displayed three main patterns. The first pattern represented no, or very subtle, osseointegration and comprised a transient increase up to a level of up to 40 Nmm and a subsequent level RTQ value (Figure 2a). The second pattern was a gradual increase up to 80 Nmm or more and a gradual leveling (Figure 2b). The pattern was interpreted as osseointegration in a plastic, partly mineralized bone. The third pattern was an initial increase followed by a break at 20–40 Nmm followed by a short level period. Thereafter, the RTQ value increased again and finally levelled out (Figure 2c). Many of the graphs also displayed a slight vibration, visualized as double curves.

At day 1, RTQ could only be attributed to friction and not to osseointegration and RTQ never exceeded 13 Nmm (7.6 ± 3.5 Nmm). There was no significant difference between Cerament sites and controls showing that the set Cerament™ did not add RTQ strength.

At 6 weeks, the specimens could be categorized into two groups according to RTQ values. Eight specimens, four Cerament™ and four controls, increased to a first peak after which there was a short decline and subsequently a level RTQ plot reaching a maximum of 36.7 ± 7.4 Nmm. The first peak was interpreted as a breakage in the attachment. The remaining eight specimens displayed a significant increase in RTQ up to a maximum of 93.5 ± 23.0 Nmm. Four of these specimens did, and four did not, display a clear initial break.

At 12 weeks, all specimens reached at least 80 Nmm with a maximum RTQ of 128.4 ± 45.5 for Cerament™ sites and 121.2 ± 30.6 for controls. Eleven of the RTQ plots displayed a clear peak.

### 2.2. Histology and Histomorphometry

**Figure 3:**
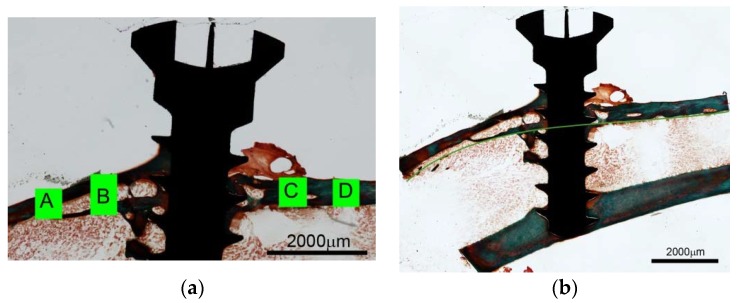
(**a**) Illustration of measuring frames around titanium screw implanted in rat tibia without Cerament™. Frame A is closest to the proximal growth plate. Ground section, Goldner stain; (**b**) Titanium screw implanted in rat tibia without Cerament™. An imaginary base line is extrapolated from the residual distal portion (right) of the inner cortical curvature. Ground section, Goldner stain.

In many specimens the original cortical medial base line was fairly resorbed and in some specimens, the base line had disappeared so that the cortex had been translocated to a peripheral position. This was primarily observed in the experimental zone next to the tibial growth plate (frames A and B) (Figure 3a).

A fan of thin trabeculae radiated from the growth plate making it somewhat difficult to define the outline of the inner cortical bone surface at times. Therefore, an imaginary inner boundary was extrapolated from the inner border of the cortex at the distal side (Figure 3b).

**Figure 4:**
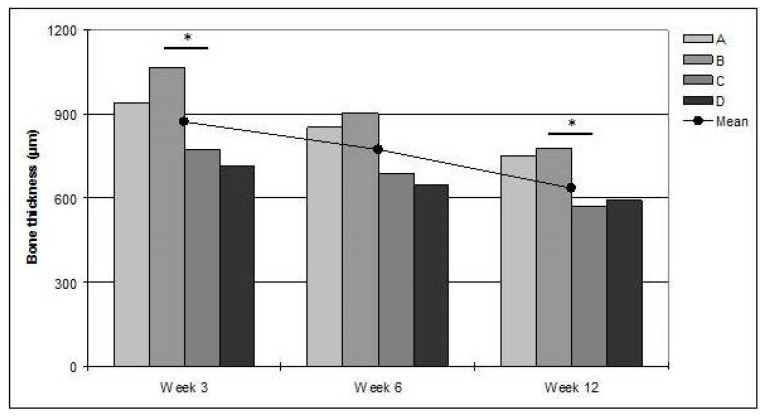
The bars denote mean bone thickness within each measuring frame (A–D). The line denotes mean thickness of all frames. * significant difference (*p* < 0.05).

After a maximum at 3 weeks (874 ± 213 μm), the mean bone thickness decreased at 6 (772 ± 128 μm), and further at 12 weeks (635 ± 73 μm; *p* < 0.05; Figure 4). The bone was thicker at the proximal than at the distal side of the titanium screw, measured from the imaginary base line. One outlier displayed an extreme bone thickness of approximately 2400 μm at 12 weeks. This animal was excluded from the analysis. The maximum cortical bone thickness was significantly greater at the proximal than at the distal side, at 12 weeks (*p* < 0.05).

**Figure 5:**
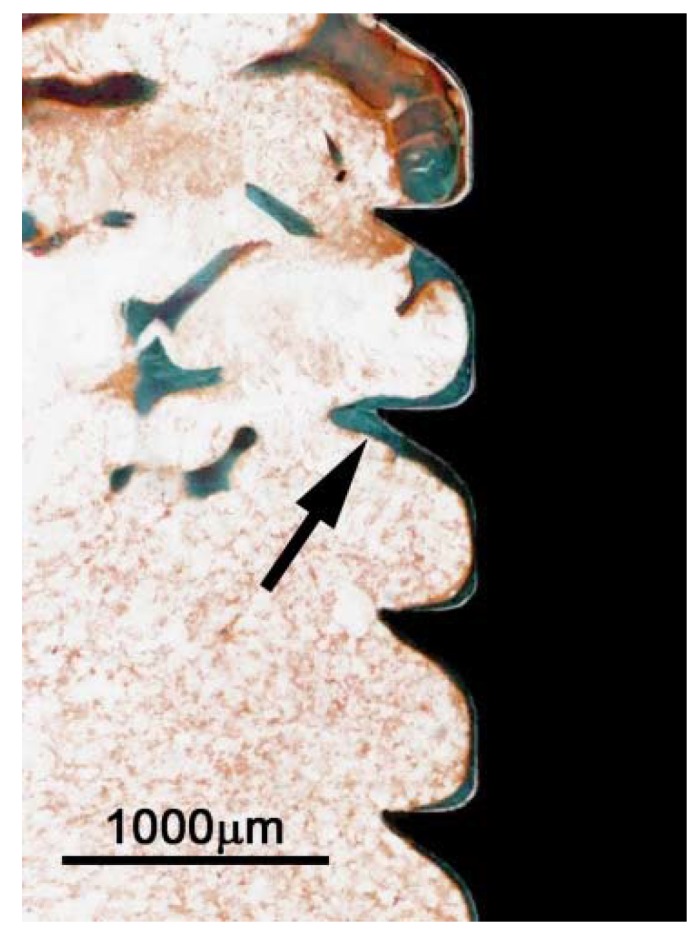
Titanium screw implanted in rat tibia for 12 weeks. Section is from an area within the original bone marrow and shows thin sheath of new bone (arrow) adjacent to the implant. Ground section, Goldner stain.

In all samples, the titanium screw was well integrated in the cortical bone and a thin sheath of bone covered the screw-threads (Figure 5). At three weeks, there was a high degree of bone remodeling on the cortical surface with an intricate network of woven trabecular bone. At 6 weeks, the trabeculae of the new bone were less elaborate and at 12 weeks, the newly formed bone had an appearance close to that of the original cortex.

In many samples, the new bone tended to creep up along the titanium screw on the proximal side. This was not observed on the distal side, rendering a statistically significant difference in the bone thickness between the frames B and C at 3 and 12 weeks (*p* < 0.05; Figure 4).

### 2.3. Discussion

Truedsson, *et al.* have previously studied how Cerament™ may conduct bone growth from a cortical bone surface [21]. The main purpose of the present study was to reveal whether the application of bone substitute would also enhance the retention of a titanium implant screw. The degree of osseointegration may to some extent vary between different implants with different surface properties [22]. In the present study, we have used a rather simple type of titanium screw but since they were evidently osseointegrated, we believe our measurements to adequately mirror the contribution of Cerament™ to the osseointegration of titanium implants in a wider perspective.

There appears to be no difference between uni- and bi-cortical implant anchorage as measured by pull-out tests [23], but it is known that removal torque tests can be higher in bi-cortical anchorage [24]. Therefore, we have tried to obtain a uni-cortical anchorage by implanting the titanium screws only 3.5 mm, since the posterior cortex of the rat tibia is V-shaped, making it difficult to achieve a standardized bi-cortical anchorage.

Inserting a titanium implant into bone initiates a cascade of events resulting in remodeling and new bone formation [25]. It has previously been shown that new bone is formed after a flap operation but after an initial growth phase, the newly formed bone will be almost completely resorbed after 12 weeks [21] whereas bone supported by Cerament™ will not be resorbed significantly during this period. As would be expected, the torque resistance of the implanted screws in the present study increased over time as they were osseointegrated but interestingly, there was no difference in RTQ between screws embedded in Cerament™ and screws inserted in bone only. This is in contrast to recently published data showing that Cerament™ increases the attachment of titanium knee prostheses in a rabbit model, as measured by pull-out force after 12 weeks [26]. However, the pull-out force resulted in a breakage of the bone-to-implant attachment whereas our findings indicate that the failure in attachment restricting RTQ was largely attributed to a breakage in the surrounding bone and not in the attachment between bone and implant [27]. Also, it has been shown that RTQ correlate to bone-to-implant contact ratio [28] and in our study, the contact area between the titanium screws and the supra-cortical new bone was rather small compared to the contact area of the implanted portion of the screw. Hence, the newly formed, and not fully matured, bone within the Cerament™ onlay on the cortical surface did not add significant strength to the attachment compared to the original cortical bone. Cerament™ in itself most probably did not contribute to the retention either.

After an initial increase of the bone thickness at three weeks, the thickness subsequently decreased although there was still a residual enlargement at 12 weeks. At the same time, there was remodeling and maturation of the trabecular bone structure. A significant difference in cortical bone thickness between frames B and C was observed at 12 weeks. There was also a difference between maximum thickness values at 12 weeks. Often, newly formed bone had “climbed” up the proximal side of the screw whereas the bone at the distal side of the screw was at level with the surrounding bone. Similar results have been reported by De Riu, *et al*. [11] who, in sheep legs, showed that significantly more bone matrix was formed on the proximal side of implant screws perpendicular to the load tension lines. A plausible explanation is that the axial load through the leg induces remodeling of the newly formed bone and resorption of the inner aspect of the former cortex, moving the proximal cortex outwards. The screw will redistribute the load between the anterior and posterior distal bone so that the distal new bone will not be induced to grow further and will instead be resorbed to the original cortical outline. As would be expected, the difference was most pronounced at 12 weeks.

## 3. Experimental Section

The study was approved by the regional Ethics committee of Lund University (M8-06), Sweden.

The experiment was made with Cerament™ (lot: G07-08-084, Bone Support AB, Lund, Sweden), a ceramic bone substitute consisting of 60% medical grade α-calcium sulphate hemihydrate (CSH; CaSO_4_·½H_2_O) and 40% sintered hydroxyapatite (HA; Ca_10_(PO_4_)_6_(OH)_2_). 0.25 g Cerament™ powder was mixed with 63 μL liquid Omnipaque™ (Iohexol 180 mg/mL, Amersham Health AS, Oslo, Norway) in a sterile petri dish yielding a putty at an L/P ratio of 1:4. 

### 3.1. Experimental Animals

#### 3.1.1. Surgical Protocol for Biomechanical Analysis

**Figure 6:**
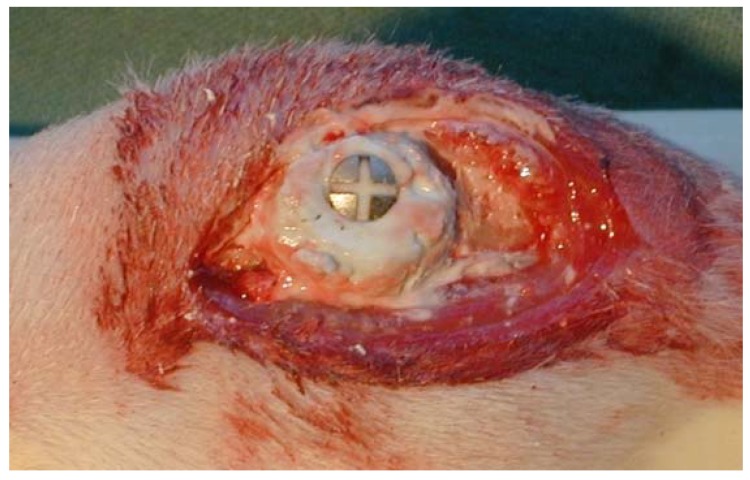
Cerament™ putty positioned around titanium screw implanted in the anterior aspect of a rat tibia.

Twenty-four male Sprague-Dawley rats (315–360 g) were anesthetized with a peritoneal injection of Pentobarbital (60 mg/mL) and Diazepam (5 mg/mL) in NaCl (0.15 M). In both hind legs, an incision was made in the proximal portion of the tibia and a periosteal flap was moved to the side. A hole with a depth of 5 mm × 1.5 mm Ø was drilled 5 mm beneath the tibial proximal growth plate. With the aid of a 3 mm guider, a titanium screw (2 mm Ø × 7 mm long, Ti-Cross drive screws, KLS Martin, Jacksonville, FL, USA) was screwed 3.5 mm into the bone in order to attain a uni-cortical anchorage without interference from the posterior cortical bone. After screw implantation, Cerament™ putty was applied around the screw on the tibial bone surface (Figure 6) in one randomly selected hind leg.

The operation field was kept dry to avoid blood contamination. The working time of the material was approximately 3 min and the setting time was 5 min. On the contra-lateral tibia, the titanium screw was left without Cerament™. Subsequently, the periosteal flaps were sutured in two layers. The animals received antibiotics preoperatively 0.05 mL Streptocilline^®^ vet (250 mg/mL + 200 mg/mL; Boehringer, Ingelheim, Germany) and postoperative analgesia 0.15 mL Temgesic^®^ (1:10; 0.3 mg/mL; Schering-Plough, Kenilworth, NJ, USA). The rats were sacrificed in groups of eight after 1 day, and 6 and 12 weeks.

#### 3.1.2. Surgical Protocol for Histology and Histomorphometry

Nine male Sprague-Dawley rats (315–360 g) were operated essentially as described above. The titanium screws were inserted 4 mm and the screws were stable, protruding 3 mm from the bone surface. The screws were covered by the periosteal flaps, sutured in two layers. The animals received antibiotics preoperatively and postoperative analgesia. The rats were sacrificed with a peritoneal injection of Pentobarbital in groups of three at 3, 6, and 12 weeks.

### 3.2. Biomechanical Analysis

Specimens of the tibiae measuring 2 cm with implants were resected and freed from soft tissue. The specimens were fixed with acrylate cement in a holder and were then kept fresh, covered by saline gauze. The specimen holder was mounted in a custom clamping device that allowed for three-dimensional adjustment. The device was fixed on an Instron 8511 load frame (High Wycombe, UK) with a MTS TestStar II controller (Minneapolis, MN, USA) equipped with a 20 Nm torque cell.

A screwdriver (KLS Martin, Jacksonville, FL, USA) made specifically for the titanium screw threads, was connected to the torque cell on the Instron/MTS machine. The RTQ was analyzed by performing a counter-clockwise rotation at the rate of 0.1 deg/sec. A torsional moment with a transversal force and minimal axial force was applied to the screw. Data was collected at 20 Hz and was obtained by turning the screwdriver 30°. The resulting RTQ curve was analyzed to determine the maximum RTQ which was used for data analysis.

### 3.3. Histology and Histomorphometry

Sections of the tibiae measuring 2 cm were dissected and fixed in 4% buffered formalin for seven days. The samples were dehydrated in ascending concentrations of ethanol, and embedded in methylmethacrylate resin (Merck, Whitehouse Station, NJ, USA). The bone specimens were cut in the middle of their longitudinal axis, through the center of the titanium screw with a 0.15 mm diamond circle saw (Isomet 11-1180, Buehler Ltd., Lake Bluff, IL, USA). Two ground-sections were prepared from each embedded specimen, polished by hand to a thickness of 50–75 μm. The sections were stained according to Goldner.

The specimens were scanned at 4× objective magnification in a light microscope (Nikon Eclipse 80i, Tokyo, Japan) equipped with a motorized stage (Scan, Märzhäuser, Wetzlar, Germany) and digital camera (Nikon DS-2Mv, Tokyo, Japan). Bone remodeling was evaluated with image analysis software (Nikon NIS-elements, BR 3.1), in a computer equipped with a scroll tablet (Trust Int. BV, Dordrecht, The Netherlands). 

The inner outline of the original cortex was resorbed in several specimens. In order to estimate the total bone height, an imaginary base line was therefore extrapolated from the residual distal portion of the inner cortical curvature (Figure 3b).

The bone thickness, expressed both as maximum thickness and average thickness within four reading frames, was measured in the experimental zones of the tibial surface. The maximum bone thickness was measured in two horizontal measuring zones of approximately 3000 μm proximally and distally to the titanium screw.

The average thickness from the inner cortical surface, or the imaginary base curvature, was measured in four frames (A–D), approx. 540 μm wide (Figure 3a).

The procedure was repeated by the same observer three times at separate occasions and the result was expressed as the mean value of the three observations. The average height of the bone in each frame was calculated as:

(1) $$average\;bone\;height_{frame}=\;\frac{frame\;area}{frame\;width}$$

### 3.4. Statistical Analysis

A statistical computer software program (SPSS) was used to perform Kruskal-Wallis, Jonckheere-Terpstra, and Wilcoxon’s signed ranks tests. Data is given as mean ± standard deviation.

## 4. Conclusions

In conclusion, Cerament™ is capable of conducting bone growth from a cortical bone surface and also to help maintain the augmented bone. However, the newly formed bone in this application does not significantly add to the osseointegrative strength of the implant screw, as measured by torque resistance, during the first 12 weeks.
